# Skin Involvement in Primary Systemic Amyloidosis

**DOI:** 10.4084/MJHID.2013.005

**Published:** 2013-01-02

**Authors:** Susheel Kumar, Rimi Som Sengupta, Nandita Kakkar, Aman Sharma, Surjit Singh, Subhash Varma

**Affiliations:** 1 Department of Internal Medicine, Postgraduate Institute of Medical Education and Research, Chandigarh, India; 2 Department of Histopathology, Postgraduate Institute of Medical Education and Research, Chandigarh, India

## Abstract

Primary systemic amyloidosis is a rare disease. It primarily involves kidney, heart, peripheral nerves and liver. Intracutaneous hemorrhage manifesting in the form of petechiae, purpura and ecchymoses due to infiltration of blood vessel walls by amyloid deposits are the most common skin lesions. We report a case of primary systemic amyloidosis with multiple, non-itchy, papular lesions in lower eyelids and lower chest wall bilaterally. Diagnosis was confirmed in this case by biopsy of skin lesions using congo red staining. Papular eruptions as seen in index patient are relatively uncommon form of skin manifestations.

## Introduction

Amyloidosis is a disease caused by extracellular deposition of insoluble polymeric protein fibrils in tissues and organs.^1^–^3^ This disease is classified as localized or systemic amyloidosis depending on whether amyloid deposition is localized to one organ system or multiple organs. Amyloid is deposited in previously apparently normal skin, with no evidence of deposits in any of internal organs in primary localized cutaneous amyloidosis (PLCA). The various types of PLCA are: more common macular, papular types and; the rare nodular form.^4^–^5^ Nodular form has been shown to progress to systemic form of amyloidosis. The recent descriptive studies have noted that rate of progression of nodular form to systemic amyloidosis is actually much lower than the 50% rate quoted in the literature in past.^6^ Skin may also be involved in systemic amyloidosis. Systemic amyloidosis is classified into primary, secondary and familial. Primary systemic amyloidosis (AL amyloidosis) may be idiopathic or myeloma-associated. It is the amyloidosis composed of immunoglobulin light chains. It involves kidney, heart, liver, peripheral nerves, autonomic nervous system and sometimes lungs.^1^–^8^ Skin involvement may be seen in AL amyloidosis. Cutaneous manifestation depends upon the site of amyloid deposition.^2^ Skin involvement other than those related to intracutaneous haemorrhage manifesting in the form of petechiae, purpura and ecchymoses due to infiltration of blood vessel walls by amyloid deposits is not very common.^2^,^9^ Here we are presenting a case of AL amyloidosis with skin involvement in the form of papular eruptions.

## Case summary

**Figure 1:**
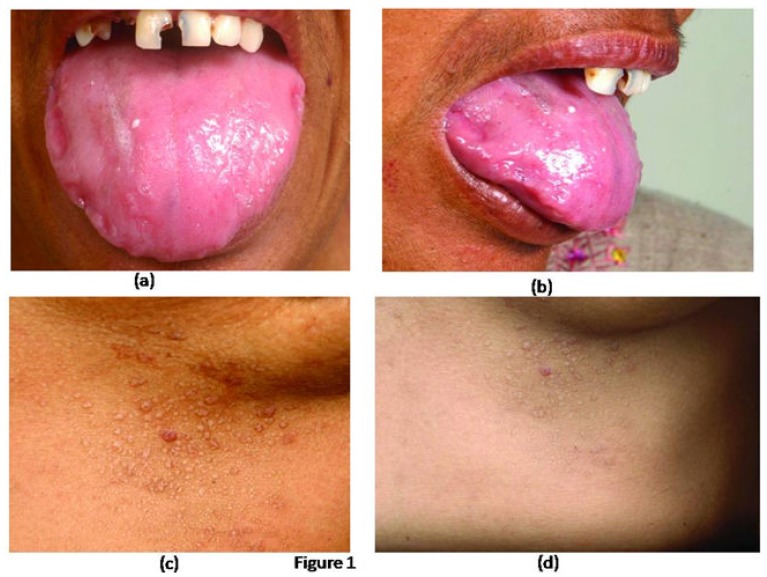
Photographs showing (a) enlarged tongue with (b) teeth marks over the lateral margin and (c, d) multiple, papular lesions over lower chest wall

**Figure 2:**
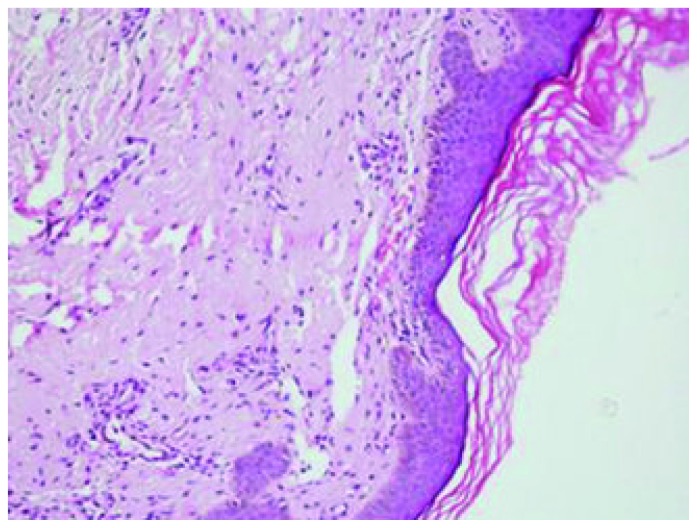
Microphotograph showing pink eosinophilic homogenous material in the dermis, H&E X 20

**Figure 3:**
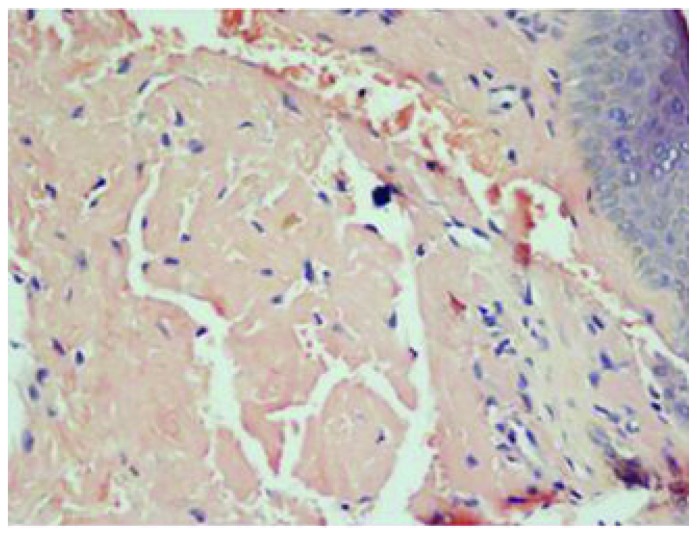
Pink eosinophilic material showing pale orange positivity with Congo red stain X 400

A 45 years old female presented with generalized weakness, easy fatiguability, along with progressive exertional shortness of breath and awareness of increase in the size of the tongue. She also noticed skin lesions over eyelids and on lower chest. On evaluation, she had macroglossia [Figure 1(a, b)]. There were multiple, non-itchy, papular lesions in lower eyelids and lower chest wall bilaterally [Figure 1(c, d)]. Cardiac auscultation revealed presence of RVS_4_. Other system examination revealed no abnormality. Hemogram and biochemical parameters were within normal limits. Urine routine and microscopic examination was also normal. 2-D Echocardiography showed concentrically thickened ventricles, diastolic dysfunction on doppler and increased echogenicity of the myocardium; overall findings suggestive of restrictive physiology with normal left ventricular systolic function. Abdominal fat pad aspiration was negative for amyloid deposits. A biopsy from skin lesions over chest wall showed pink acellular eosinophilic homogenous material in the dermis on haemotoxylin & eosin staining (Figure 2). This pink eosinophilic material showed pale orange positivity with congo red staining consistent with the diagnosis of amyloidosis (Figure 3). There was persistence of KMnO4 staining suggesting diagnosis of primary amyloidosis. Urine and serum protein electrophoresis as well as serum immunofixation was negative. Free light chain assay was within normal limits –free kappa−15.8mg/ml (Normal range: 3.3–19.4 mg/ml), free lambda −16.4mg/ml (Normal range: 5.7–26.33mg/ml). Bone Marrow biopsy showed 10% plasma cells. She was started on chemotherapy (Melphalan, prednisolone and thalidomide). Two weeks after discharge, she presented with right sided weakness. Computed tomography head showed acute infarct in left basal ganglia and internal capsule. She was managed conservatively and discharged.

## Discussion

Cutaneous manifestation in AL amyloidosis depends upon the site of amyloid deposition.^2^ Superficial dermal deposition of amyloid produces shiny waxy translucent papules. Flexural areas are sites of predilection, including the eyelids, retroauricular region, neck, axillae, inframammary area, umbilicus, inguinal and anogenital regions. Lesions may also be found on the central face, lips, tongue and buccal mucosa.^2^ Our patient had multiple skin colored papules over lower eyelid and chest wall in inframammary area. A biopsy from skin lesions over chest wall was consistent with the diagnosis of amyloidosis showing deposits of material which were positive for Congo red staining. There was persistence of KMnO4 staining suggesting diagnosis of primary amyloidosis.^10^,^11^ Other rare cutaneous alterations seen in AL amyloidosis are: hyperpigmentation, infiltrate similar to scleroderma, alopecia areata or universal, nail dystrophies, cutis laxa and lesions similar to *cutis verticis girata* in the scalp.^12^–^14^ Macroglossia as seen in index patient is pathognomic of AL amyloidosis and is seen in around 10% of patients.^15^

Subcutaneous abdominal fat aspiration, the preferred method for detecting systemic amyloidosis, has a sensitivity of 80%.^16^,^17^ In index case abdominal fat pad aspiration was negative for amyloid deposits. The various reasons for false negative results of this diagnostic test in index case could be: insufficient amount of material, inadequate staining technique, improper use of polarizing instruments, and insufficient light intensity. Therefore, in case of negative findings in the fat aspirate from a patient with a persistently high clinical suspicion of amyloidosis or progressive disease for which there is no other explanation, fat aspiration should be repeated, and the aspirate should be examined by a experienced cytopathologist. Biopsy is also very important for the diagnosis. Hematoxylin and eosin staining suggests the possibility of amyloidosis but Congo red staining confirms the diagnosis. Congo red staining results in a brick red color of amyloid when seen under ordinary light and under polarized light shows classical green birefringence.^1^–^3^ Serum protein electrophoresis reveals a spike pattern in around half of patients with primary AL amyloidosis. Two-thirds of patients with AL amyloidosis show monoclonal protein on immunoelectrophoresis of serum and urine respectively. The frequency of patients with an identifiable monoclonal protein rises to about 86% on screening of both serum and urine together.^2^ Diagnostic sensitivity improves further on combining immunofixation on agarose gel electrophoresis and bone marrow plasma cell light chain ratio analysis.^18^ Nevertheless, in some cases with the clinical features of AL amyloidosis it is not possible to demonstrate a paraprotein, as was noted in index patient as well.^19^

This case demonstrates uncommon type of skin manifestations in the form of papules over lower eyelid and chest wall. A skin biopsy from these lesions will substantiate the diagnosis of amyloidosis as was seen in index case.

## References

[1] Falk RH, Comenzo RL, Skinner M (1997). The systemic amyloidoses. N Engl J Med.

[2] Breathnach SM, Burns T, Breathnach S, Cox N, Griffiths C (2004). Metabolic and Nutritional Disorders. Rook’s Textbook of Dermatology.

[3] Lachmann HJ, Hawkins PN, Wolff K, Goldsmith LA, Kat SI, Gilchrest BA, Paller AS, Leffell DJ (2008). Amyloidosis and the Skin. Fitzpatrick’s Dermatology in General Medicine.

[4] Fernandez-Flores A (2012). Cutaneous amyloidosis: a concept review. Am J Dermatopathol.

[5] Schreml S, Szeimies RM, Vogt T, Landthaler M, Schroeder J, Babilas P (2010). Cutaneous amyloidoses and systemic amyloidoses with cutaneous involvement. Eur J Dermatol.

[6] Kalajian A, Waldman M, Knable AL (2007). Nodular primary localized cutaneous amyloidosis after trauma: a case report and discussion of the rate of progression to systemic amyloidosis. J Am Acad Dermatol.

[7] Kyle RA, Gertz MA (1995). Primary systemic amyloidosis: clinical and laboratory features in 474 cases. Semin Hematol.

[8] Agarwal A, Singla S, Bansal M, Nair B (2012). Bilateral Pleural Effusions due to Pulmonary Amyloidosis as the Presenting Manifestation of Multiple Myeloma. Mediterr J Hematol Infect Dis.

[9] Silverstein SR (2005). Primary, systemic amyloidosis and the dermatologist where classic skin lesions may provide the clue for early diagnosis. Dermatol Online J.

[10] Van Rijswijk MH, van Heusden CW (1979). The potassium permanganate method. A reliable method for differentiating amyloid AA from other forms of amyloid in routine laboratory practice. Am J Pathol.

[11] Janssen S, Elema JD, van Rijswijk MH, Limburg PC, Meijer S, Mandema E (1985). Classification of amyloidosis: immunohistochemistry versus the potassium permanganate method in differentiating AA from AL amyloidosis. ApplPathol.

[12] Gerster JC, Landry M, Dudler J (2000). Scleroderma-like changes of the hands in primary amyloidosis. J Rheumatol.

[13] Hunt SJ, Caserio RJ, Abell E (1991). Primary systemic amyloidosis causing diffuse alopecia by telogen arrest. Arch Dermatol.

[14] Prat C, Moreno A, Vigas M, Jucgls A (2008). Nail dystrophy in primary systemic amyloidosis. J EurAcadDermatolVenereol.

[15] Murthy P, Laing MR (1994). Macroglossia BMJ.

[16] Van Gameren II, Hazenberg BP, Bijzet J, van Rijswijk MH (2006). Diagnostic accuracy of subcutaneous abdominal fat tissue aspiration for detecting systemic amyloidosis and its utility in clinical practice. Arthritis Rheu.

[17] Westermark P, Davey E, Lindbom K, Enqvist S (2006). Subcutaneous fat tissue for diagnosis and studies of systemic amyloidosis. Acta Histochem.

[18] Perfetti V, Garini P, Vignarelli MC (1995). Diagnostic approach to and follow-up of difficult cases of AL amyloidosis. Haematologica.

[19] Crow KD (1977). Primary amyloidosis. Br J Dermatol.

